# Genetic study of the hepcidin gene (*HAMP*) promoter and functional analysis of the c.-582A > G variant

**DOI:** 10.1186/1471-2156-11-110

**Published:** 2010-12-10

**Authors:** Silvia Parajes, Arturo González-Quintela, Joaquín Campos, Celsa Quinteiro, Fernando Domínguez, Lourdes Loidi

**Affiliations:** 1 Fundación Pública Galega de Medicina Xenómica, Santiago de Compostela, Spain; 2 Department of Internal Medicine, Hospital Clínico Universitario, Santiago de Compostela, Spain; 3 Department of Physiology, Universidad de Santiago de Compostela, Santiago de Compostela, Spain

## Abstract

**Background:**

Hepcidin acts as the main regulator of iron homeostasis through regulation of intestinal absorption and macrophage release. Hepcidin deficiency causes iron overload whereas its overproduction is associated with anaemia of chronic diseases. The aims of the study were: to identify genetic variants in the hepcidin gene (*HAMP*) promoter, to asses the associations between the variants found and iron status parameters, and to functionally study the role on *HAMP *expression of the most frequent variant.

**Results:**

The sequencing of *HAMP *promoter from 103 healthy individuals revealed two genetic variants: The c.-153C > T with a frequency of 0.014 for allele T, which is known to reduce hepcidin expression and the c.-582A > G with a 0.218 frequency for allele G. In an additional group of 224 individuals, the c.-582A > G variant genotype showed no association with serum iron, transferrin or ferritin levels.

The c.-582G *HAMP *promoter variant decreased the transcriptional activity by 20% compared to c.-582A variant in cells from the human hepatoma cell line HepG2 when cotransfected with luciferase reporter constructs and plasmid expressing upstream stimulatory factor 1 (USF1) and by 12-14% when cotransfected with plasmid expressing upstream stimulatory factor 2 (USF2).

**Conclusions:**

The c.-582A > G *HAMP *promoter variant is not associated with serum iron, transferrin or ferritin levels in the healthy population. The *in vitro *effect of the c.-582A > G variant resulted in a small reduction of the gene transactivation by allele G compared to allele A. Therefore the effect of the variant on the hepcidin levels *in vivo *would be likely negligible. Finally, the c.-153C > T variant showed a frequency high enough to be considered when a genetic analysis is done in iron overload patients.

## Background

Hepcidin was first isolated from urine as an antimicrobial peptide [1] and thereafter, it has been shown to act as the main regulator of iron homeostasis through regulation of intestinal iron absorption and macrophage iron release [2-5]. The first evidence was provided by mice studies which showed increased hepcidin mRNA levels in the liver of iron overload mice [6]. Further animal models and human disorders confirmed the relationship between this peptide and iron. Genetically modified mice showed that animals with reduced hepcidin expression developed severe iron overload [7], while those with increased expression had severe iron deficiency anaemia at birth [8]. In humans, inactivating mutations of hepcidin result in a rare form of juvenile haemochromatosis [9], whereas hepcidin overexpression in inflammation causes anaemia of chronic diseases with features of iron restricted erythropoiesis [10]. Besides, hepcidin has been described as a modifier peptide that exacerbates the phenotypic expression of patients with hereditary haemochromatosis (HFE) homozygous for the common *HFE *gene mutation, p.Cys282Tyr [11-13].

The gene encoding hepcidin, *HAMP*, is mainly expressed in the liver in situations of iron overload [14]. It is upregulated by hemojuvelin [15], transferrin receptor 2 [16] and HFE protein [17] mediated through the bone morphogenetic protein (BMP) signalling pathway [18]. Hepcidin is also upregulated in inflammation by interleukin 6 (IL-6) and other cytokines through the Janus kinase/signal transducer and activator of transcription (JAK/STAT) signalling pathway [6,10,19]. Hepcidin is inhibited by hypoxia [20] and augmented erythropoiesis [21]. More recently, matriptase-2 has been described as the first known negative regulator of hepcidin expression [22].

Lately, two genetic variants in the *HAMP *promoter have been described as modulators of iron overload. Firstly, the c.-582A > G variant has been associated with higher liver iron concentration and with higher serum ferritin levels in beta-thalassemic patients [23]. Secondly, the c.-153C > T mutation, which is located within a BMP-Responsive Element, has been shown to contribute to a severe phenotype in HFE related haemochromatosis [11].

The aim of the study was to identify genetic variants in the *HAMP *promoter and their frequency distribution in the Galician population. Another objective was to assess possible associations between these variants and the serum iron, serum transferrin and serum ferritin levels in a random sample of Galician probands. Finally, we performed functional *in vitro *studies to determine the effect of variants on *HAMP *expression.

## Results

### Identification of genetic variants in the hepcidin gene promoter

The sequencing of the *HAMP *promoter in the random sample of 103 healthy individuals allowed the identification of two genetic variants each with an allele frequency higher than 0.01. Therefore, both loci could be considered polymorphic. The first variant c.-582A > G was found in 49 promoter sequences (58 individuals were homozygous for allele A, 41 heterozygous AG and 4 homozygous GG), which resulted in an allele frequency of 0.762 for c.-582A and 0.218 for c.-582G. This variant corresponded to single nucleotide polymorphism (SNP) rs10421768 in the NCBI SNP database http://www.ncbi.nlm.nih.gov/snp/. The second variant c.-153C > T was found in only three heterozygous CT. Thus allele frequency of allele c.-153T was 1.4%. Furthermore, unique variants were found in two heterozygous individuals: c.-572C > T and c.-188C > T. No transcription factor binding sites were predicted for these unique variants http://www.biobase-international.com/pages/index.php?id=transfac.

### c.-582A > G genotype and its relation with biochemical parameters

The genotype frequencies of the SNP located 582 bp upstream did not differ significantly between our sample of 224 individuals and the 103 healthy probands initially studied. No significant differences were found in the levels of serum iron, serum transferrin, transferrin saturation or ferritin levels between the c.-582A > G variant genotypes (Table 1). No significant differences were found either when males and females were stratified (data not shown).

**Table 1:** Serum iron, serum transferrin, serum ferritin and transferrin saturation according to HAMP c.-582A > G genotype in Galician population

	c.-582A > G Genotype	
	
	A/A	A/G and G/G
**N (%)**	128 (57.1)	96 (42.9)

**SI mean ± SD (μg/dL)**	55.6 ± 30.7	55.5 ± 31.2

**ST mean ± SD (mg/dL)**	265.7 ± 28.0	263.6 ± 30.8

**SF mean ± SD (μg/dL)**	95.9 ± 73.7	106.6 ± 74.5

**TS mean ± SD (%)**	15.0 ± 8.5	15.2 ± 8.8

SI: Serum iron, ST: serum transferrin, SF: serum ferritin, TS transferrin saturation

### Transactivation assays

**Figure 1:**
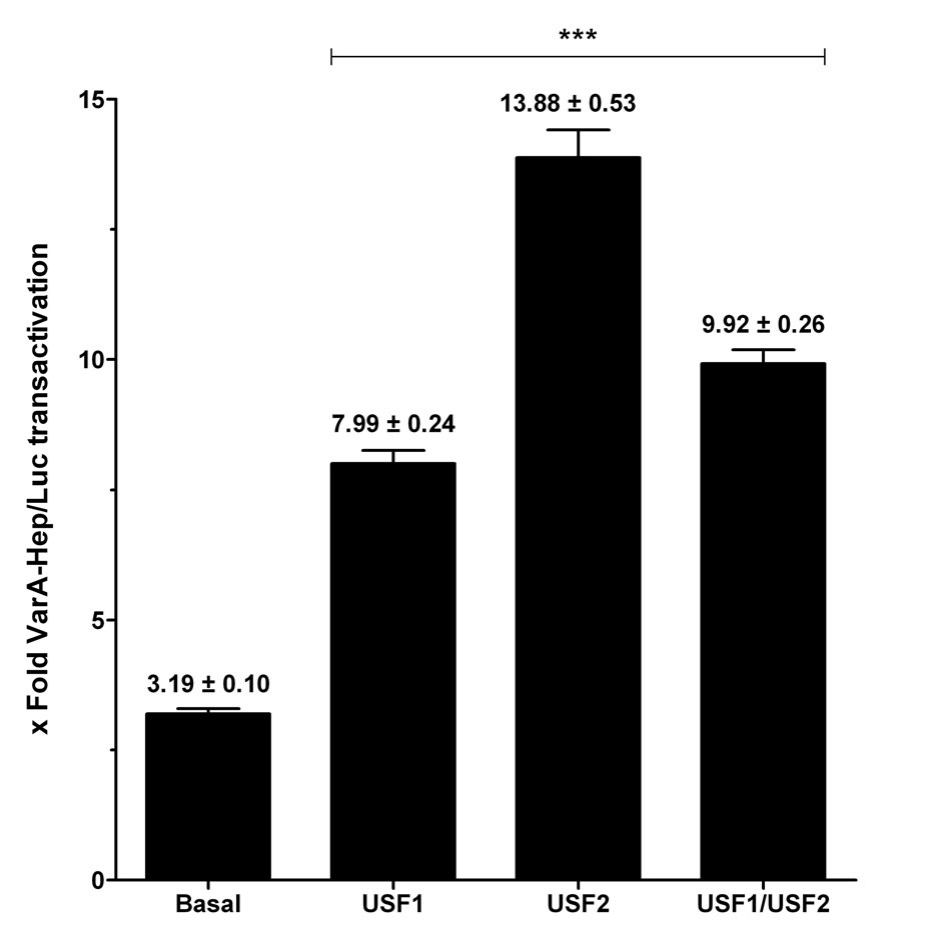
**Functional analysis of the c.-582A variant**. Cotransfection of HepG2 cells with pCMV-USF1, pCMV-USF2 or pCMV-USF1+ pCMV-USF2 (1:1) increased the promoter activation compared to cells transfected with VarA-Hep/Luc constructs alone (basal). Data represent means of three independent experiments (± SEM) ***p < 0.001

**Figure 2:**
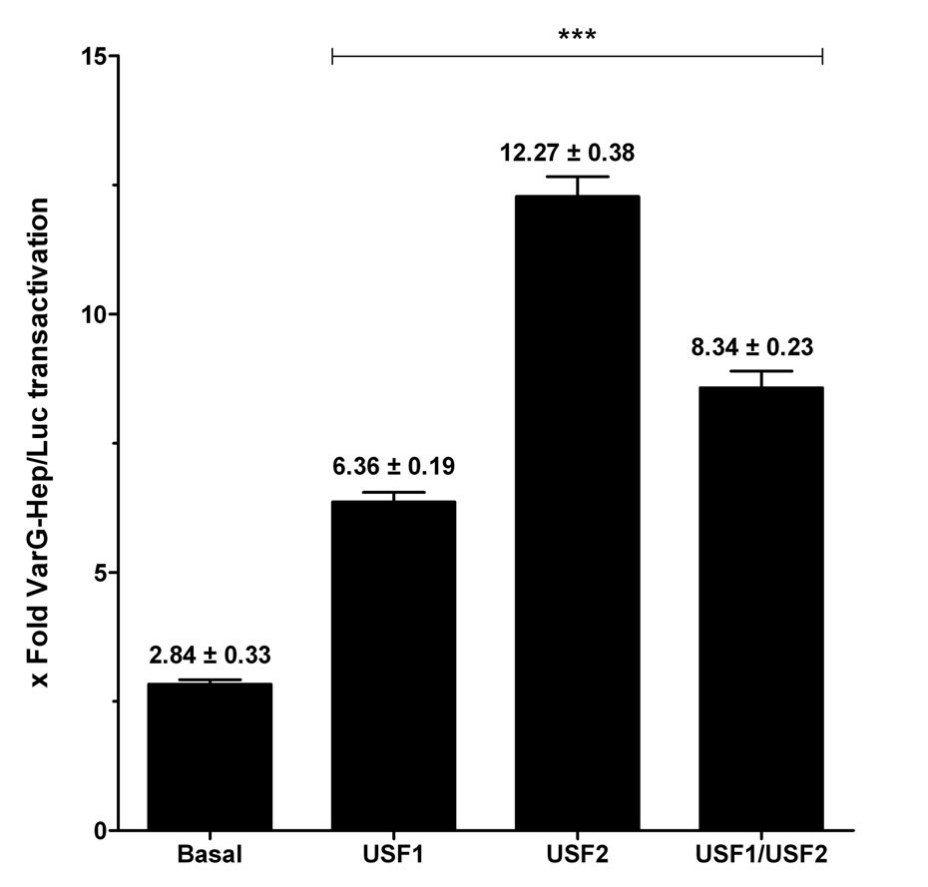
**Functional analysis of the c.-582A variant**. Cotransfection of HepG2 cells with pCMV-USF1, pCMV-USF2 or pCMV-USF1+ pCMV-USF2 (1:1) increased the promoter activation compared to cells transfected with VarG-Hep/Luc constructs alone (basal). Data represent means of three independent experiments (± SEM) ***p < 0.001

The fold activation of VarA-Hep/Luc construct was 3.19 ± 0.10 in basal conditions and it increased to 7.99 ± 0.24 when cells were cotransfected with pCMV-USF1, 13.88 ± 0.53 with pCMV-USF2 and 9.92 ± 0.26 with pCMV-USF1+ pCMV-USF2. These results indicated that either USF1, USF2 or the combination of both (USF1+USF2 (1:1)) can transactivate VarA-Hep/Luc construct with a 2.5 ± 0.3, 4.3 ± 0.7 and 3.1 ± 0.3 (mean ± SEM) fold induction, respectively (Figure 1). The same experiments carried out for the VarG-Hep/Luc construct gave similar results. The fold activation of the VarG-Hep/Luc construct in basal conditions was 2.84 ± 0.33 and it increased to 6.36 ± 0.19 when cotransfected with pCMV-USF1, 12.27 ± 0.38 with pCMV-USF2 and 8.34 ± 0.23 with pCMV-USF1+ pCMV-USF2 (1:1 ratio). These meant a 2.2 ± 0.3, 4.3 ± 0.5 and 3.0 ± 0.4 (mean ± SEM) fold induction driven by USF1, USF2 or USF1+USF2 (1:1), respectively (Figure 2).

**Figure 3:**
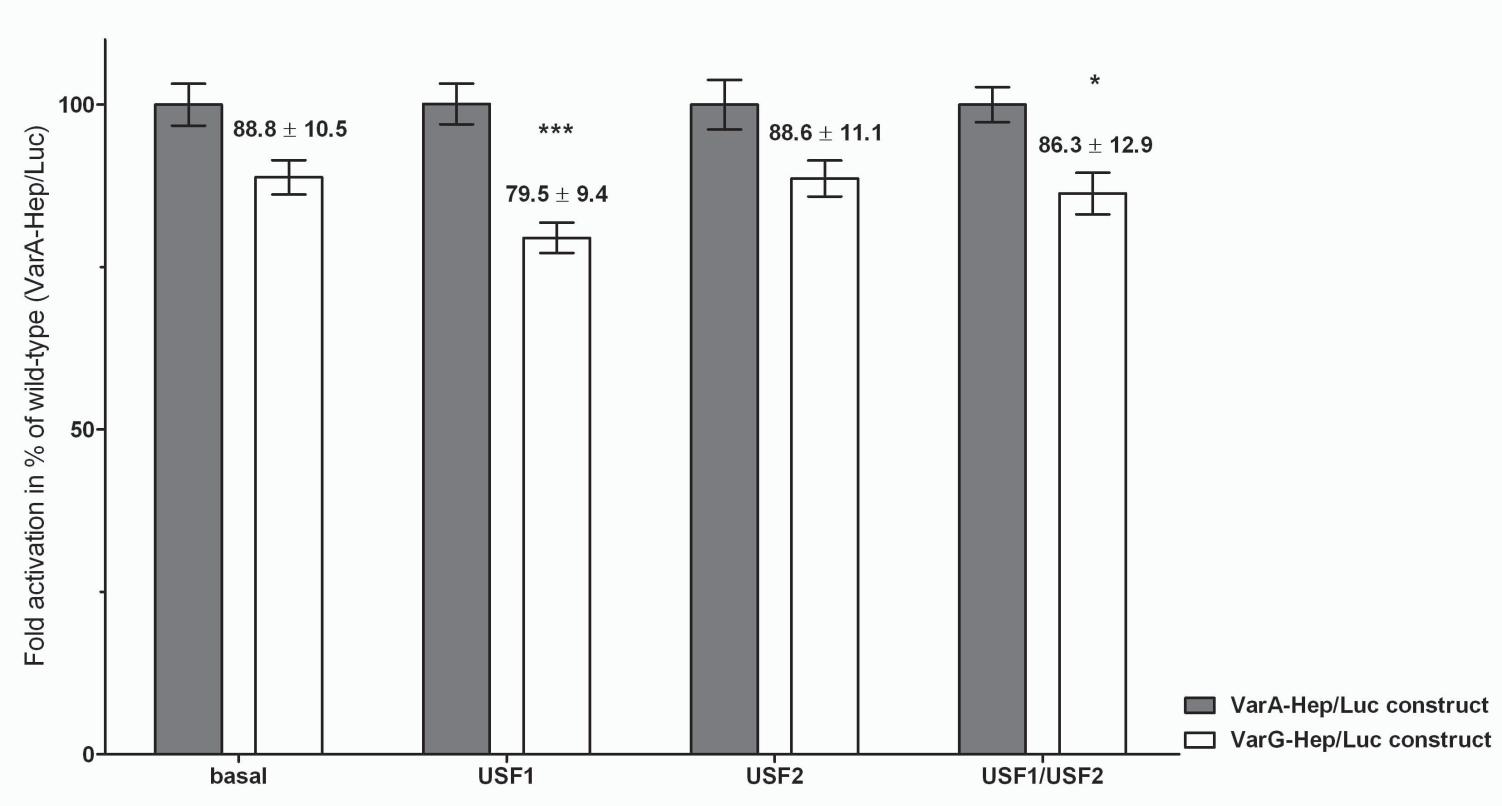
**The c.-582A > G variant decreases hepcidin promoter transcription activity**. HepG2 cells were transfected with VarA-Hep/Luc or VarG-Hep/Luc constructs alone (basal) and cotransfected with pCMV-USF1, pCMV-USF2 or pCMV-USF1+ pCMV-USF2 (1:1). Data represent means of three independent experiments (± SEM) ***p < 0.001, *p < 0.05

The c.-582G variant slightly decreased *HAMP *promoter transcriptional activity. Basal luciferase activity from VarG-Hep/Luc construct was 88.8% compared to that of VarA-Hep/Luc construct. When those constructs were transactivated with USF1, the VarG-Hep/Luc showed a significant 20% decrease in activity compared to VarA-Hep/Luc (*p *< 0.001). When transactivated with USF2 and USF1+USF2 a 12-14% reduction was obtained for the VarG-Hep/Luc with respect to VarA-Hep/Luc (*p *> 0.05 and *p *< 0.05, respectively) (Figure 3).

## Discussion

Hepcidin is the main regulator of iron homeostasis and, therefore, a better understanding of its regulation is of great interest. In the present work, we sequenced, in an initial sample group, the promoter of the hepcidin gene, *HAMP*. This allowed the identification of two genetic polymorphic variants in our population. In a further group of individuals the c.-582A > G was genotyped and a possible relationship with biochemical iron status markers was studied. In addition, we performed functional-in-vitro analysis to characterize the effect of the c.-582A > G variant on the hepcidin gene transactivation.

In the present work, we first sequenced the promoter of the hepcidin gene *HAMP *in a group of 103 healthy individuals. This allowed the identification of two polymorphic sites. In another group of 224 individuals, the c.-582A > G variant was genotyped and its possible relationship with biochemical data as well as its effect on *HAMP *transactivation was studied. The frequency distribution of this SNP in the Galician population is similar to that of a cohort of Italian healthy donors [24] and furthermore to those of other European populations http://www.ncbi.nlm.nih.gov/snp/.

The c.-582A > G variant is located within an E-box, which is a responsive element for helix-loop-helix transcription factors (bHLH-ZIP). The canonical sequence of the E-box is CACGTG (consensus CANNTG). When the A is substituted by G, the E-box would be abolished and unable to bind the transcription factors. USF1 and USF2 are bHLH-ZIP transcription factors that have been shown to positively regulate hepcidin expression through E-boxes within the promoter [25]. Therefore, we decided to study the in-vitro effect of the c.-582A > G variant on the *HAMP *transactivation in response to USF stimulation. Our results confirmed the ability of USF1 and USF2 to transactivate the *HAMP *promoter in HepG2 cells. Additionally, we found that the USF transactivation was maintained in the c.-582G promoter. However, the USF promoter transactivation was slightly reduced in the case of the c.-582G allele compared to c.-582A. The subtle decrease in transactivation might be caused by the fact that only one of the four E-boxes present in the promoter is affected. Nonetheless, it can not be ruled out that different endotoxin content or slightly incorrect DNA estimation in the plasmid preparations caused the small differences found between the two variants.

No significant association between the c.-582A > G genotype and serum iron, serum transferrin, transferrin saturation or ferritin levels were found, which might reflect no differences in liver iron concentration. These results are in agreement with Bruno and col. that did not find association either [24]. Conversely, this association was found among a series of thalassemia major patients. The experiments performed in the HepG2 cells showed, at its maximum, a 20% transactivation reduction by the c.-582G allele, compared to c.-582A. In addition, one has to bear in mind that the *in-vitro *model used may not completely reflect what happens *in-vivo*. This data together with the fact that *HAMP *is regulated by many different signals [6,10,15-18,20-22] suggested that the effect of the c.-582A > G polymorphism on hepcidin levels, and therefore on the iron status *in-vivo *is most likely negligible. We hypothesize that the c-.582A > G variant in the human *HAMP *promoter would have no effect on the hepcidin transcription in normal situations but it might have some effect in physiopathological situations where more hepcidin was needed. However, further studies would be necessary to confirm this hypothesis.

The variation at the second polymorphic site, c.-153C > T, in the promoter region of the hepcidin gene had been previously identified in a patient with massive iron overload homozygous for the p.Cys282Tyr mutation in *HFE *[11]. Island and col. demonstrated that the c.-153C > T mutation, which is located in a BMP-Responsive Element, reduced basal hepcidin gene expression and impaired its response to BMPs and IL-6. This variation has been found at a very low frequency by other investigators: Island and col. did not found it in 200 chromosomes from healthy volunteers [11], Barton and col. found a frequency of 0.06% among 785 Haemochromatosis and Iron Overload Screening (HEIRS) Study participants [26]. Both groups concluded that the variation in haemochromatosis patients is not worth to study. On the other hand, Aguilar-Martinez and col. found the variant with a 5% frequency in a group of haemochromatosis patients without the p.Cys282Tyr mutation in *HFE*, whilst in healthy individuals of the same population the allele frequency was only 0.4%. They suggested that this variant should be studied in a selected iron overload patients [27]. Our finding of a 1.4% frequency of the c.-153T allele in the Galician population additionally supports this recommendation for genotyping this locus in iron overload Galician patients.

## Conclusions

Due to the role of hepcidin as a key modulator of systemic iron levels, it is plausible that differences in the level of its expression may partly account for the phenotypic variant in iron metabolism between individuals. In this report, no association was found between serum iron, transferrin or ferritin levels and the c.-582A > G *HAMP *promoter variant in a healthy population. This result was supported by the functional *in-vitro *studies, which showed only a 10-20% reduction of the promoter transactivation by allele c.-582G compared to the c.-582A allele.

## Methods

### Sample population

A total of 327 individuals were used for the present study. Initially, a sample of 103 healthy individuals was recruited from the university students and employees of the Hospital Clínico Universitario at Santiago de Compostela. Information about their parents' and grandparents' geographic origins was provided and all were from the Spanish Galician region, which is a relatively isolated European population at the westernmost continental edge. A second independent random sample of 224 individuals was randomly recruited from the general adult population of the Galician town La Estrada. Serum ferritin levels of this group were between 10-300 ng/mL. Detailed description has been reported elsewhere [28]. Informed consent was obtained from each participant. All the procedures were conducted according to the principles expressed in the Declaration of Helsinki and to the ethical standards of the local Ethics Committee.

### Biochemical determinations

Serum iron and serum transferrin were assayed in an ADVIA-2400 Chemistry System (Siemens Medical Diagnostics, Germany) and serum ferritin was measured in an ADVIA-Centaur Immunoassay System (Siemens). Transferrin saturation (TS) was calculated with the following formula: TS (in %) = [serum iron (in μg/dL) × 71]/serum transferrin (in mg/dL). With these methods, normal concentrations in adults are as follows: serum iron, 45-160 μg/dL (males) and 40-150 μg/dL (females); serum transferrin, 215-365 mg/dL (males) and 250-380 mg/dL (females); transferrin saturation, 7-53%; and serum ferritin 10-295 ng/mL (males) and 25-325 ng/mL (females).

### Genetic analysis of the *HAMP *promoter

Blood samples were collected and DNA was extracted from peripheral blood leucocytes following standard procedures. A fragment of 951bp that extended from nucleotide 1013 upstream the ATG translation initiation codon of *HAMP *to 9bp downstream of exon 1 was PCR amplified with the following pair of primers: forward 5'-GTACTCATCGGACTGTAGATGTTAGC-3' and reverse 5'-GTGACAGTCGCTTTTATGGGGCCTGC-3'. PCR products were purified and both strands were directly sequenced using the BigDye terminator kit and run in the 3730 }l DNA analyzer (Applied Biosystems, Foster City, CA, USA).

### c.-582A > G variant genotype

The rs10421768 polymorphism located 582 bp upstream the ATG translation initiation codon was genotyped using the TaqMan^® ^functionally tested assay c_2604942_10 in a 7300 Real-Time PCR system (Applied Biosystems).

### Cell culture

The human hepatoma cell line HepG2 was obtained from the European Cell Culture Collection (ECACC, UK) and maintained in Dulbecco's modified media (DMEM) with 4500 mg/L glucose supplemented with 10% foetal bovine serum, 2 mM glutamine, 50 units/mL penicillin, 50 μg/mL streptomycin and 100 μg/mL neomycin.

### Plasmids

Two 951 bp fragments, which included 942bp of the human *HAMP *promoter plus 9 bp of 5'UTR of exon 1 were PCR amplified from homozygous c.-582A and c.-582G genomic DNAs respectively (Forward 5'-GGCTCGAGGTACTCATCGGACTGTAGATGTTAGC-3', Reverse 5'-GGAAGCTTGTGACAGTCGCTTTTATGGGGCCTGC-3').

PCR products were double-digested with HindIII and XhoI restriction enzymes (Promega Corporation, Madison, WA, USA). VarA-Hep/Luc and VarG-Hep/Luc constructs were generated using T4 DNA ligase (Promega) by cloning digested products into the same restriction sites of pGL3-Control vector (Promega). The nucleotide sequence of the constructs was confirmed by DNA sequencing.

Plasmids pCMV-USF1 and pCMV-USF2, (kindly provided by Dr. M. Sawadogo University of Texas, MD USA and Dr. H K. Bayele University College London UK), were used for transactivation assays.

### Luciferase assays

Approximately 1.4 × 10^5 ^HepG2 cells were plated in 12-well plates 24 h before transfection. Cells were transiently transfected with 1000 ng of DNA using FugeneHD transfection reagent at an 8:2 ratio according to manufacturer's protocol (Roche Applied Sciences, Indianapolis, USA). For promoter transactivation assays cells were transiently cotransfected with 350 ng of Hep/Luc constructs and 450 ng of pCMV-USF1, pCMV-USF2 or the combination of these latter two plasmids at a 1:1 ratio. In all cases, 200 ng of pRL-TK vector (Promega) was included as an internal control of transfection efficiency. When necessary, pcDNA3.1 empty vector was used to equalize the total amount of DNA. pGL3-Control vector was used in each experiment as an interassay control to normalize transfection efficiencies. Cells were incubated with transfection reagents for 48 h at 37°C in full DMEM media, followed by further 18 h incubation in fresh full media. Luciferase and Renilla activities were determined using Dual-Glo^®^Luciferase Assay System (Promega) according to manufacturer's protocol. Luminiscence was measured in a TRIAD LT luminometer (Dynex Technologies, Chantilly, VA, USA). All the experiments were independently performed in triplicate.

Transactivation of the *HAMP *promoter is expressed as fold induction of luciferase/renilla luminescence ratio of Hep/Luc expressing cells over the ratio obtained from cells transfected with pGL3-Control vector. Data are presented as mean values ± SEM.

### Statistical analysis

Statistical analysis was performed using SPSS analysis software v.16.0 (*SPSS *Inc., Chicago, IL, USA). The differences found in the values of the biochemical indicators of iron status among the various genotypes at position c.-582 were tested by the Mann-Whitney and Kruskal-Wallis tests. The results from Luciferase assays were compared using one-way ANOVA for repeated measurements followed by Tukey's posthoc test. These analyses were performed using GraphPad Prism v.5.0 (GraphPad Software, San Diego, California). In all cases a *p *value less than 0.05 was considered significant.

## Authors' contributions

SP participated in the conception and design of the study, carried out the functional studies and helped to draft and revised the manuscript. AGQ made acquisition of data for the study, carried out statistical analysis and revised the manuscript. JC made acquisition of data for the study. CQ carried out the molecular genetic studies. FD participated in the design of the study, interpretation of data and revised the manuscript. LL Conceived and designed the study and wrote the manuscript. All authors read and approved the final manuscript.
